# Identification of expression quantitative trait loci by the interaction analysis using genetic algorithm

**DOI:** 10.1186/1753-6561-1-s1-s69

**Published:** 2007-12-18

**Authors:** Junghyun Namkung, Jin-Wu Nam, Taesung Park

**Affiliations:** 1 Bioinformatics Program at College of Natural Science, Seoul National University, San 56-1, Sillim-dong, Gwanak-gu, Seoul 151-747 Korea; 2 Department of Statistics, Seoul National University, 56-1 Shillim-Dong, Kownak-Gu, Seoul 151-747 Korea

## Abstract

Many genes with major effects on quantitative traits have been reported to interact with other genes. However, finding a group of interacting genes from thousands of SNPs is challenging. Hence, an efficient and robust algorithm is needed. The genetic algorithm (GA) is useful in searching for the optimal solution from a very large searchable space. In this study, we show that genome-wide interaction analysis using GA and a statistical interaction model can provide a practical method to detect biologically interacting loci. We focus our search on transcriptional regulators by analyzing gene × gene interactions for cancer-related genes. The expression values of three cancer-related genes were selected from the expression data of the Genetic Analysis Workshop 15 Problem 1 data set. We implemented a GA to identify the expression quantitative trait loci that are significantly associated with expression levels of the cancer-related genes. The time complexity of the GA was compared with that of an exhaustive search algorithm. As a result, our GA, which included heuristic methods, such as archive, elitism, and local search, has greatly reduced computational time in a genome-wide search for gene × gene interactions. In general, the GA took one-fifth the computation time of an exhaustive search for the most significant pair of single-nucleotide polymorphisms.

## Background

Gene expression variation usually results in phenotypic variation such as disease status. Searching for the regulators of gene expression is important to understand the mechanism of phenotypic change or individual variation. In recent studies, Morley et al. [1] have identified expression quantitative trait loci (eQTL), which contain transcriptional regulators nearby the tested markers. Additionally, they identified genomic regions containing master transcription regulators, which influenced multiple genes' expression. However, their method has only accounted for the marginal effect of the regulators. Note that the interactions among genes are common phenomena in complex biological systems. For example, *Apolipoprotein E *(*ApoE*), an important predictor of coronary artery disease, was discovered to interact with the unlinked *low-density lipoprotein receptor *(*LDLR*) gene in determining plasma cholesterol levels [2].

Because relatively inexpensive high-throughput genotyping techniques are available, genome-wide association studies using single-nucleotide polymorphisms (SNPs) are becoming popular. However, finding a candidate locus from thousands of SNPs accounting for gene × gene interaction is one of the greatest challenges. A search for all possible interactions requires $\sum_{k}n!/(k!(n-k)!)$ evaluations, where *k *is the number of SNPs in an interaction model and *n *is the total number of SNPs.

One practical approach to dealing with enormous numbers of evaluations is a two-stage analysis in which the first stage selects the major SNPs with marginal effects and the second stage identifies significant interactions between the selected major SNPs and the remaining SNPs [3]. However, this strategy does not cover the interactions among the SNPs without marginal effect. Thus, an efficient method is needed to search for all possible interactions with less computational burden.

In this study, we introduce a genetic algorithm (GA) to perform a genome-wide search for interactions. The GA is a learning method motivated by an analogy to biological evolution. GAs have been applied successfully to a variety of learning tasks and to other optimization problems [4,5]. For example, Carlborg et al. [6] applied a GA in the analysis of quantitative trait loci (QTL) interaction. In order to improve the performance of the GA, we added several strategies such as archive, elitism, two distinct mutations, and local search in the vicinity.

Using the Genetic Analysis Workshop 15 (GAW15) Problem 1 data set, we investigated whether the identification of interactions among eQTL contributes to the construction of a biological network for the cancer-related genes. We first identified eQTL by single SNP analysis, and then identified additional eQTL by the interaction analysis using our GA. The genetic effect was assessed by the analysis of variance (ANOVA) model with no specific Mendelian inheritance model assumed. The eQTL identified by interaction analysis may be candidate components to reconstruct genetic architecture (relationship) among genes responsible for the phenotype, such as cancer in this study. Finally, we compared the time-complexity between our GA and an exhaustive search algorithm on the basis of the number of evaluations.

## Methods

### Data

The GAW15 Problem 1 data set contains genome-wide scan genotypes and gene expression data from lymphoblastoid cells. They were collected from the 14 Utah CEPH (Centre d'Etude du Polymorphisme Humain) three-generation pedigrees. Genotypes of 2882 SNPs from autosomal and X-linked loci and expression values of 3554 genes for all individuals were provided. Among the 2882 SNPs, 190 SNPs were monomorphic, so they were excluded from the analysis.

We used data from the 56 founders to find eQTL that are associated with the expression levels of the three major cancer-related genes: *BRCA1*, *TAF15*, and *EP300*. The mutation of *BRCA1 *is known as a causal factor for the breast and ovarian cancer; *EP300 *for colorectal, breast, pancreatic cancer, and acute lymphocytic leukemia; *TAF15 *for acute lymphocytic leukemia.

### Association test for the detection of eQTL using ANOVA models

Two types of ANOVA models were used. The response variable was the expression value of the cancer-related genes. The first type of ANOVA model contained only main effects and was applied to test for the association between a given SNP and a corresponding gene expression level. The second type of ANOVA model contained main effects for two SNPs and an interaction effect between them; it was used to test for the interaction effect between two SNPs on the gene expression values.

### Implementation of genetic algorithm

**Figure 1:**
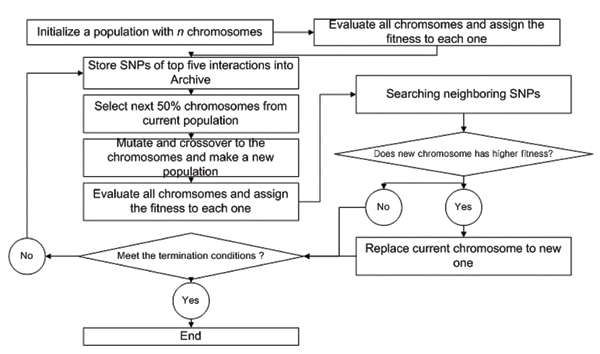
Flow chart of genetic algorithm.

We first introduce GA terms. A chromosome is a set of variables which constitutes a fitness function. A set of chromosomes is called population. The population size is the number of chromosomes selected for one generation (iteration). The fitness is a function that assigns an evaluation score for a chromosome. An archive stores the variables in chromosomes showing the top ranked by fitness during the searches. The GA process is depicted in Figure 1.

#### 1. Initialization

A chromosome consists of one SNP pair (one major and one minor, see below) to be searched for a significant interaction. The initial population is constructed by choosing a predefined *n *major and minor SNP pairs, where a major SNP is randomly selected from a list of marginally significant SNPs detected by the first type of ANOVA models and a minor SNP is randomly selected from a list of marginally non-significant SNPs. The boundary between the major and minor SNPs is a cross-over point.

#### 2. Fitness function

For the evaluation of each chromosome, we fit the second type of ANOVA model and use -(log(*p*-values)) as fitness measures for the interaction terms.

#### 3. Selection method

After the evaluation of the chromosomes, all pairs of SNPs are sorted according to the fitness measure. The top 50% are selected as parents to reproduce in the next generation. To guarantee a stable convergence, we use elitism. The elites (a user-defined number) are inherited by the next generation without any variation.

#### 4. Genetic operators

We use two different genetic operators, mutation, and cross-over. The first genetic operator, mutation, changes a SNP to a different SNP with a given mutation rate. A major SNP mutation replaces the major SNP by arbitrary one in the archive, a temporary storage containing unique SNPs in the top five ranks of fitness. Through mutation of major SNPs, a minor SNP in the archive can become major SNPs after second generation. Thus, interactions among minor SNPs are also searched. A minor SNP mutation alters the minor SNP to another minor SNP. The second genetic operator, cross-over, exchanges two minor SNPs among two randomly selected chromosomes.

#### 5. Local search

In order to find additional significant interactions within the linkage disequilibrium (LD) block, we perform a local search by examining SNPs in the vicinity (+1 and -1) of all SNPs implicated in a significant interaction. If the fitness increases, the SNP under evaluation is replaced by the local test SNP. After the local search, the population is sorted again.

## Results

### Identification of eQTL by the interaction analysis

We focused our search on eQTLs for three cancer related-genes. We tested the marginal effects of 2692 SNPs by fitting the first type of ANOVA model with individual SNPs and the expression values of the three cancer-related genes. The SNPs showing *p*-values of less than 0.001 were considered to have marginal effects and were classified as major SNPs in the genetic algorithm. All remaining SNPs were classified as minor SNPs in the genetic algorithm. Using this set of major and minor SNPs, we applied the GA for interaction analysis. We used a major mutation rate of 0.4, a minor mutation rate of 0.1, a cross-over rate of 0.6, and a population size of 1000.

For the individual SNP (marginal effects) analysis, four eQTLs for *BRCA1*, three eQTLs for *EP300*, and 18 eQTLs for *TAF15 *were identified as having marginal effects at the 0.001 significance level (*α*). Next, we applied the GA to identify significant pairs of SNPs. To maintain a nonimal significance level of 0.05, then the Bonferroni correction for multiple testing requires *p *< 1.38 × 10^-8^. WIth this threshold in mind, we chose to evaluate three values of *α *(1 × 10^-6^, 1 × 10^-7^, and 1 × 10^-8^). The GA yielded the following number of significant SNP pairs for these values of *α*: seven, three, and zero significant pairs for *BCRA1*; nine, zero, and zero pairs for *EP300*; and 1284, 319, and 50 pairs for *TAF15*.

**Figure 2:**
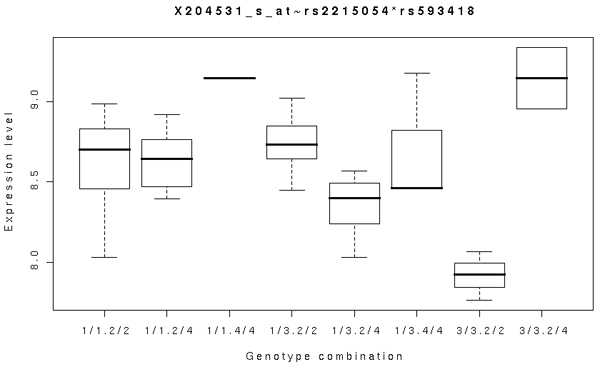
**Box plot of the expression of *BRCA1 *for eachgenotype combination of rs2215054 and rs593418**. When the first SNP has 3/3 genotype, the expression level increases as the second genotype is changed from 2/2 to 2/4. When the first SNP has 1/1 or 1/3 genotype the expression level decreases, which illustrates the presence of interaction between the first and second SNPs on the expression levels.

The GA identified SNP pairs that do not have individual marginal effects. For example, rs2215054 and rs593418 showed a significant interaction effect on *BRCA1 *gene expression (*p*-value = 4.01 × 10^-7^). Figure 2 shows that the individuals doubly homozygous for the minor alleles have the lowest level of gene expression, perhaps indicating a recessive-recessive interaction between the two loci. Because both loci lacked marginally significant effects on the response level, it cannot be detected by the two-stage approach. Another example of an interaction between SNPs lacking marginal effects includes rs1545610 and rs946437 (*p*-value = 4 × 10^-6^). We note that rs946437 is located in the UTR region of the estrogen-related receptor gamma (*ESRRG*) gene.

The eQTLs identified in the interaction analysis can be further investigated. For example, 65 unique eQTLs were identified for *TAF15 *from out of the 50 significant SNP pairs (*p *< 1 × 10^-7^). Eleven of these eQTLs are located in genic regions. Additionally, we examined whether functional groups are overrepresented in the eQTLs by gene ontology (GO) analysis using GOstat [7] to identify overrepresented elements within the 11 gene-containing eQTLs. GOstat performs Fisher's exact test assuming the hypergeometric distribution. In our analysis of the eQTL regions, the corresponding genes significantly overrepresented protein-binding functions (*p *= 0.038). This illustrates how statistical interaction analysis can provide a useful clue to study the biological interaction.

### Comparison of genetic algorithm and exhaustive search

We first analyzed SNPs for the presence of linkage disequilibrium (LD) blocks. When an LD block is defined by D' > 0.8 across consecutive SNPs, the block had two SNPs on average. This LD block size information was used in the local search portion of the GA.

In order to compare the time-complexity between our GA and the exhaustive search algorithm, we estimated the numbers of evaluations required to search all pairs for the most significant interaction effects. The top ten most significant SNP pairs were selected by an exhaustive search algorithm, which took 3,622,086 evaluations. Next, the number of evaluations required to detect any pair from list of the top ten pairs from the GA was computed. Table 1 shows the numbers of evaluations for ten runs of the GA, which ranged from 6,000 to 68,000, approximately one-fifth of the number of evaluations needed by the exhaustive search. Next, we fixed the number of evaluations used in the GA and then counted how many top ten significant interaction pairs were found by the GA. During 300,000 evaluations of the GA, the average number of interactions detected by the GA was six. This number of evaluations is only 8.2% of the exhaustive search (3,622,086).

**Table 1:** The number of GA evaluations to obtain one of the ten most significant interactions

	SNP1			SNP2					
					
Expressed gene	rs number	Gene	Chr	rs number	Gene	Chr	*p*-Value	Generations	Evaluations
*EP300*	rs1037782	*LOC441716*	15q13.1	rs1274224	*ZBTB20*	3q13.2	8.97 × 10^-7^	11	11000
	rs616113	*CACNA2D3*	3p21.1	rs312926	*BRUNOL5*	19p13	2.01 × 10^-6^	20/8^a^	20000/8000^a^
	rs1909160		12	rs1824780		5	2.23 × 10^-6^	28/7/37^a^	28000/7000/37000^a^
	rs2219328		3	rs721602		12	2.46 × 10^-6^	7	7000
	rs1909160		12	rs1463234		3	4.33 × 10^-6^	9/46^a^	9000/46000^a^
	rs627953		18	rs1537375	*LOC401495*	9	9.89 × 10^-6^	15	15000
									
*BRCA1*	rs1037782		15	rs1274224	*ZBTB20*	3q13.2	8.97 × 10^-7^	26/27/11/21^a^	26000/27000/11000/21000^a^
	rs1025048		5	rs1430509		4	4.33 × 10^-7^	22/12^a^	22000/12000^a^
	rs1861478		12	rs1391708		12	7.47 × 10^-7^	46	46000
	rs2215054	*KIAA0672*	17p12	rs179562		14	8.15 × 10^-7^	6	6000
	rs2215054	*KIAA0672*	17p12	rs593418		18	4.01 × 10^-7^	10	10000
	rs2215054	*KIAA0672*	17p12	rs179562		14	8.15 × 10^-7^	38	38000
									
*TAF15*	rs2180052		6	rs1798294		12	8.49 × 10^-9^	74/12^a^	74000/12000^a^
	rs1798294		12	rs564021		18	4.11 × 10^-9^	33	33000
	rs1798294		12	rs1480691	*SGCZ*	8p22	1.6 × 10^-9^	32	32000
	rs766083	*FER1L3*	10q24	rs2040692		22	5.53 × 10^-9^	68	68000
	rs1798294		12	rs1524163	*NR5A2*	1q32.1	5.43 × 10^-9^	12	12000
	rs1798294		12	rs1548543		19	6.21 × 10^-10^	25/11/11/40^a^	25000/11000/11000/40000^a^

^a^SNP pairs are identified from more than two runs.

## Conclusion and discussion

In this study, we applied a GA to detect eQTL interactions, which showed comparable performance to an exhaustive search with a smaller number of evaluations. The reduction in computing time becomes greater as the search space becomes larger (looking forward to the case of 500 K genome-wide genotype data). Implementation of a local search makes it possible to reduce the computation time more dramatically as marker density increases. However, the GA does not guarantee a global optimum, in general. Carlborg et al. (2000) examined this issue in a simulation study, comparing a GA with exhaustive search and a two-stage search for different types of genetic interaction models. They showed the GA has almost as high an efficiency as exhaustive search for all interaction models they examined. In practical terms, the GA can be used to provide at least suboptimal solutions with a much lower computational burden.

Genome-wide interaction analysis using the GA enables us to identify pairs of SNPs that do not have individual marginal effects. The identified SNP pairs can be further examined with previously known biological information. In the example of *TAF15 *gene, we showed that the identified loci may affect the trait through biological interactions identified in a GO analysis.

We can improve the performance of genome-wide GA searches for the higher-order SNP interactions by incorporating biological information, such as biological pathways, protein-protein interactions, and linkage results. In particular, pathway information may provide the most useful prior knowledge for reducing the computation time. As an example, there was a report on interactions of genetic variants of genes in the same pathway affecting a psychiatric disease [8].

In our study, we used only one individual per family to satisfy the independence assumption of ANOVA models. As a result, the number of subjects was not large, which may have caused a lack of power. Thus, it is desirable to develop a statistical model that accounts for family structure when testing for interactions, so that all individuals in a given data set are included in the analysis.

## Competing interests

The author(s) declare that they have no competing interests.
